# A 52 weeks dupilumab treatment for moderate to severe atopic dermatitis in Korea: long-term efficacy and safety in real world

**DOI:** 10.1038/s41598-021-02950-4

**Published:** 2021-12-07

**Authors:** Dong Hyek Jang, Seok Jae Heo, Hyung Don Kook, Dong Heon Lee, Hye Jung Jung, Mi Yeon Park, Jiyoung Ahn

**Affiliations:** 1 Department of Dermatology, National Medical Center, 245 Eulji-ro, Jung-gu, Seoul, 04564 Korea; 2 Department of Biostatistics and Computing, Yonsei University Graduate School, Seoul, Korea

**Keywords:** Immunology, Biomarkers, Skin diseases

## Abstract

Previously, we have reported short term effectiveness and safety of dupilumab in Korea. In this study, we are trying to report the long-term effectiveness and safety of dupilumab in Korea. Ninety-nine patients with moderate to severe AD were analyzed. They were evaluated using Eczema Area and Severity Index (EASI), Numerical Rating Scale (NRS), Patient Oriented Eczema Measure (POEM), and Dermatology Quality of Life Index (DLQI) at baseline, week 16, 32 and 52. Efficacy outcomes showed higher improvement at 52 weeks compared with 16 weeks; high percentual reductions in EASI (88.1%), peak pruritus NRS (65.6%), POEM (67.2%), and DLQI (69.0%) compared to baseline. Proportion of patients achieving EASI 75 and 90 were 90.2% and 53.7%. POEM and DLQI had high correlation with clinical measured outcomes. In the analysis for the factors affecting achievement of EASI 90, female gender (OR 2.5), eosinophilia (OR 0.2) and elevated LDH (OR 0.07) were significantly associated. Most frequent adverse events included facial erythema (19.2%) and conjunctivitis (17.2%), which were mild/moderate and resolved during treatment. In conclusion, dupilumab treatment for 52 weeks in Korean patients with moderate-to-severe AD confirmed long term effectiveness and safety.

## Introduction

Atopic dermatitis (AD) is a representative chronic inflammatory systemic disease^1^, and various treatments have been developed depending on the stage^2,3^. From classical systemic immunosuppressants to newly developed biologics and small molecules that inhibit various cytokines that contribute to the immune response, there are many ongoing studies^4–6^. Among them, dupilumab, a monoclonal antibody that blocks the shared receptor component for interleukin (IL)-4 and IL-13^7^, is a first biological agent approved for the treatment of patients with moderate to severe AD. Until recently, it has been most actively used and considered as a game changer in the treatment of severe AD.

Dupilumab has shown efficacy and safety in existing phase 3 and 4 clinical trials^8^ and long-term effectiveness in various real-world studies^9–14^. Previous data from Korea have shown significant improvements and safety in moderate-to-severe AD patients treated with dupilumab for 16 weeks^15^. However, data for effectiveness and safety for long-term treatment with dupilumab in AD patients in real world is limited. Therefore, we aimed to evaluate the long-term effectiveness and safety of 52 weeks of treatment with dupilumab in moderate to severe AD patients in Korea and investigate the prognostic factors affecting the treatment outcome.

## Methods

### Study design

This retrospective analysis was conducted through electronic medical records of patients with moderate to severe AD treated with dupilumab at National Medical Center in Korean from September 2018 to December 2020. Patients were assigned to dupilumab 600 mg on day 1, then to dupilumab 300 mg every 2 weeks, with the exception of 9 patients who received dupilumab 300 mg every 3 or 4 weeks. Along with dupilumab, all patients were treated with topical calcineurin inhibitor (TCI). Some patients who have been previously under treatment with systemic immunosuppressants gradually reduced dose in order to prevent abrupt discontinuation and received initially concomitant treatment with dupilumab and the previously used systemic immunosuppressants, including systemic steroids. After sufficient tapering, all concomitant treatments except dupilumab were discontinued within 2 weeks. This study was approved by the Institutional Review Board of the National Medical Center, and was conducted in accordance with the principles of the Declaration of Helsinki.

### Patients and efficacy outcomes

Eligible patients had an Eczema Area and Severity Index (EASI) score of 16 or higher and were over 18 years old who were diagnosed with AD based on the diagnostic criteria^16^ by a dermatologist. Patients were assessed for efficacy outcomes and measured for laboratory tests at baseline, 16, 32, and 52 weeks after treatment as follows: Efficacy outcomes; EASI, peak pruritus Numerical Rating Scale (NRS) , Patient Oriented Eczema Measure (POEM) , and Dermatology Quality of Life Index (DLQI)^17–20^, Laboratory tests; Whole Blood Count, Liver Function Test, Renal Pannel, Lipid Profile, Immunoglobulin E (IgE), Total Eosinophil Count (TEC), and Lactate Dehydrogenase (LDH).

### Statistical analysis

Statistical analysis was performed using R 3.6.3 version (R Foundation for Statistical Computing, Vienna, Austria). The changes in efficacy outcomes and laboratory tests between the baseline and 16, 32, and 52 weeks of treatment were analyzed using the linear mixed model with discrete time points. Correlation analysis between efficacy outcomes at 52 weeks and laboratory test results was performed to identify the factors most relevant to the patient's quality of life. The differences in efficacy outcomes after 52 weeks according to the level of IgE, TEC, and LDH at each measurement point were investigated. In addition, multivariative logistic regression analysis was performed using age, sex, disease onset, initial combined treatment, IgE, TEC, and LDH as independent variables to determine whether there are factors affecting the achievement of EASI 90 after 52 weeks of treatment. Statistical significance was determined at a p value less than 0.05.

### Informed consent

Need of informed consent is waived by the Institutional Review Board of National Medical Center.

## Results

### Demographics

Of 99 patients, 58 men and 41 women had an average age of 30.92 years; 48 (48.48%) were in their 20 s to 30 s, while 38 (38.38%) were in their 30 s and 40 s. One third of patients (36%) had a family history of allergic diseases and more than half (57.58%) had personal history of other allergic diseases. Among them, allergic rhinitis, allergic conjunctivitis and asthma were the most frequent (85.96%, 29.82%, and 21.05%, respectively). About half (55.56%) of patients had known allergies mostly caused by house dust mite (96.36%). Topical steroid was the most frequent previous treatment used (92.93%) followed by TCI (68.69%). Treatment with systemic steroid and non-steroid immunosuppressive drugs accounted for 86.89% and 61.61% of the subjects, respectively. Cyclosporine was the most common non-steroid immunosuppressant (86.89%). The proportion of oriental medicine and folk remedies were 52.53% and 27.27%, respectively, with oriental medicine in more than half of the cases (Table 1).

**Table 1:** Baseline demographics and history of treatment (n = 99).

Type	Variables	Mean (min, max) or count (%)
Demographics	Age, years	30.92 (18.00–53.00)
	10–20	4 (4.04%)
	20–30	48 (48.48%)
	30–40	38 (38.38%)
	40–50	7 (7.07%)
	50–60	2 (2.02%)
	Gender	
	Male	58 (58.59%)
	Female	41 (41.41%)
	Occupation	
	Yes	68 (68.69%)
	Marriage	
	Yes	19 (19.19%)
	Disease onset	
	Adult exacerbation	6 (6.06%)
	Childhood	93 (93.94%)
	Family history of allergy	
	Yes	36 (36.36%)
	Allergy history	57 (57.58%)
	Allergic conjunctivitis	17 (29.82%)
	Allergic rhinitis	49 (85.96%)
	Asthma	12 (21.05%)
	Known allergy	55 (55.56%)
	House dust mite	53 (96.36%)
	Mold	19 (34.55%)
	Food	5 (9.09%)
	Cat	6 (10.91%)
	Dog	3 (5.45%)
History of treatment	Topical treatment	
	Topical steroid	92 (92.93%)
	Topical calcineurin inhibitor	68 (68.69%)
	Systemic treatment	
	Systemic steroid	65 (65.66%)
	Immunosuppressant	61 (61.61%)
	Cyclosporine	53 (86.89%)
	Methotrexate	6 (9.84%)
	Mycophenolate mofetil	1 (1.64%)
	Azathioprine	1 (1.64%)
	Phototherapy	20 (20.20%)
	Immunotherapy	13 (13.13%)
	Oriental medicine	52 (52.53%)
	Folk remedy	27 (27.27%)

Most patients (82.83%) were treated with dupilumab with TCI only over the 52 weeks. For patients initiating dupilumab in combination with other systemic drug for tapering, the initial combination treatment was cyclosporine in 7%, methotrexate in 6%, and oral steroid in 4%.

### Efficacy of treatment

#### Baseline value of efficacy outcomes

Outcome measures used in this study have been validated for atopic dermatitis and are recommended by HOME^21^. The mean baseline values for EASI, pruritus NRS, POEM, and DLQI are presented in Table 2, and correspond to severe disease.

**Table 2:** Change in efficacy outcomes after 16, 32 and 52 weeks—all showed a significant decrease.

	Baseline	After 16 weeks	After 32 weeks	After 52 weeks
Mean EASI score ± SD	30.02 ± 10.84	7.61 ± 4.77*	4.66 ± 3.25*	3.50 ± 2.88*
Mean percent change ± SD in EASI		74.90 ± 11.57	84.49 ± 9.05	88.14 ± 8.56
Mean NRS score ± SD	8.37 ± 1.72	3.36 ± 2.13*	3.24 ± 2.07*	2.80 ± 1.82*
Mean percent change ± SD in NRS		59.37 ± 5.42	59.51 ± 28.93	65.55 ± 23.05
Mean POEM ± SD	23.73 ± 4.35	9.99 ± 6.33*	9.00 ± 6.19*	7.34 ± 5.86*
Mean percent change ± SD in POEM		56.56 ± 27.34	59.95 ± 29.34	67.21 ± 29.34
Mean DLQI ± SD	22.37 ± 5.27	8.93 ± 6.59*	7.60 ± 6.86*	6.54 ± 5.74*
Mean percent change ± SD in DLQI		59.59 ± 29.23	61.01 ± 29.51	69.02 ± 27.61

SD, standard deviation; EASI, Eczema Area and Severity Index; NRS, Numerical Rating Scale; POEM, patient-oriented eczema measure; DLQI, Dermatology Life Quality Index.

*p value < 0.05, P value calculated by linear mixed model with discrete time points.

#### Changes in efficacy outcomes at 16, 32 and 52 weeks

All outcome measures reduced from the most severe levels towards mild values overtime. EASI values reduced by 75% at week 16 (mean 7.6) and 88% at week 52 (mean 3.5). Pruritus NRS reduction was 59% at week 16 (mean 3.4) and 66% at week 52 (mean 2.8). POEM score (0–28) improved by 57% at week 16 (mean 10) and 67% at week 52 (mean 7.3) and DLQI improved by 60% at week 16 (mean 8.9) and 69% at week 52 (mean 6.5, representing small impact in life). All differences were statistically significant vs baseline values (p < 0.001) (Table 2).

#### EASI 75 and EASI 90 at 52 weeks

**Figure 1:**
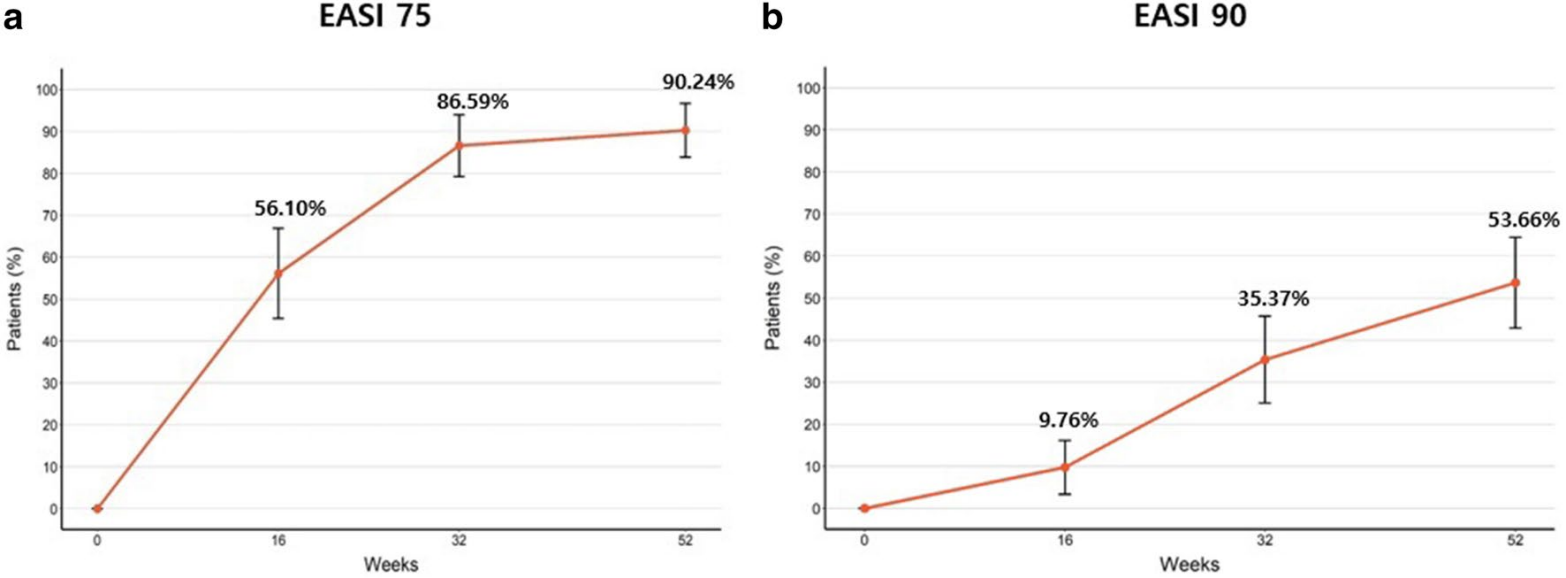
The proportion of (**a**) EASI 75 and (**b**) EASI 90 at baseline, after 16, 32 and 52 weeks.

EASI 75 was achieved by 56.10%, 86.59%, and 90.24% of patients at 16, 32, and 52 weeks, respectively, and EASI 90 was achieved in 9.76%, 35.37%, and 53.66% of patients at 16, 32, and 52 weeks, respectively (Fig. 1a,b).

#### Changes in laboratory tests at 16, 32 and 52 weeks

Compared to the baseline values, there was a significant decrease in IgE, TEC, and LDH at 16, 32, and 52 weeks. There was a 53.64% decrease for IgE from 3964.05 to 1837.87, 44.82% for TEC from 931.67 to 514.13, and 30.15% for LDH from 280.81 to 196.14 (Table 3). No significant changes in other laboratory findings were observed.

**Table 3:** Change in laboratory test after 16, 32 and 52 weeks—all showed a significant decrease.

	Baseline	After 16 weeks	After 32 weeks	After 52 weeks
Mean serum total Ig E ± SD	3964.05 ± 6411.23	2601.08 ± 3843.12*	1960.34 ± 3020.99*	1837.87 ± 3043.89*
Mean serum TEC ± SD	931.67 ± 805.49	737.54 ± 846.80*	618.93 ± 628.58*	514.13 ± 487.55*
Mean serum LDH ± SD	280.81 ± 85.32	219.19 ± 65.02*	212.12 ± 63.21*	196.14 ± 43.80*

Ig E, immunoglubulin E; TEC, total eosinophil count; LDH; lactate dehydrogenase.

*p < 0.05, p value calculated by linear mixed model with discrete time points.

### Correlations between efficacy outcomes

**Figure 2:**
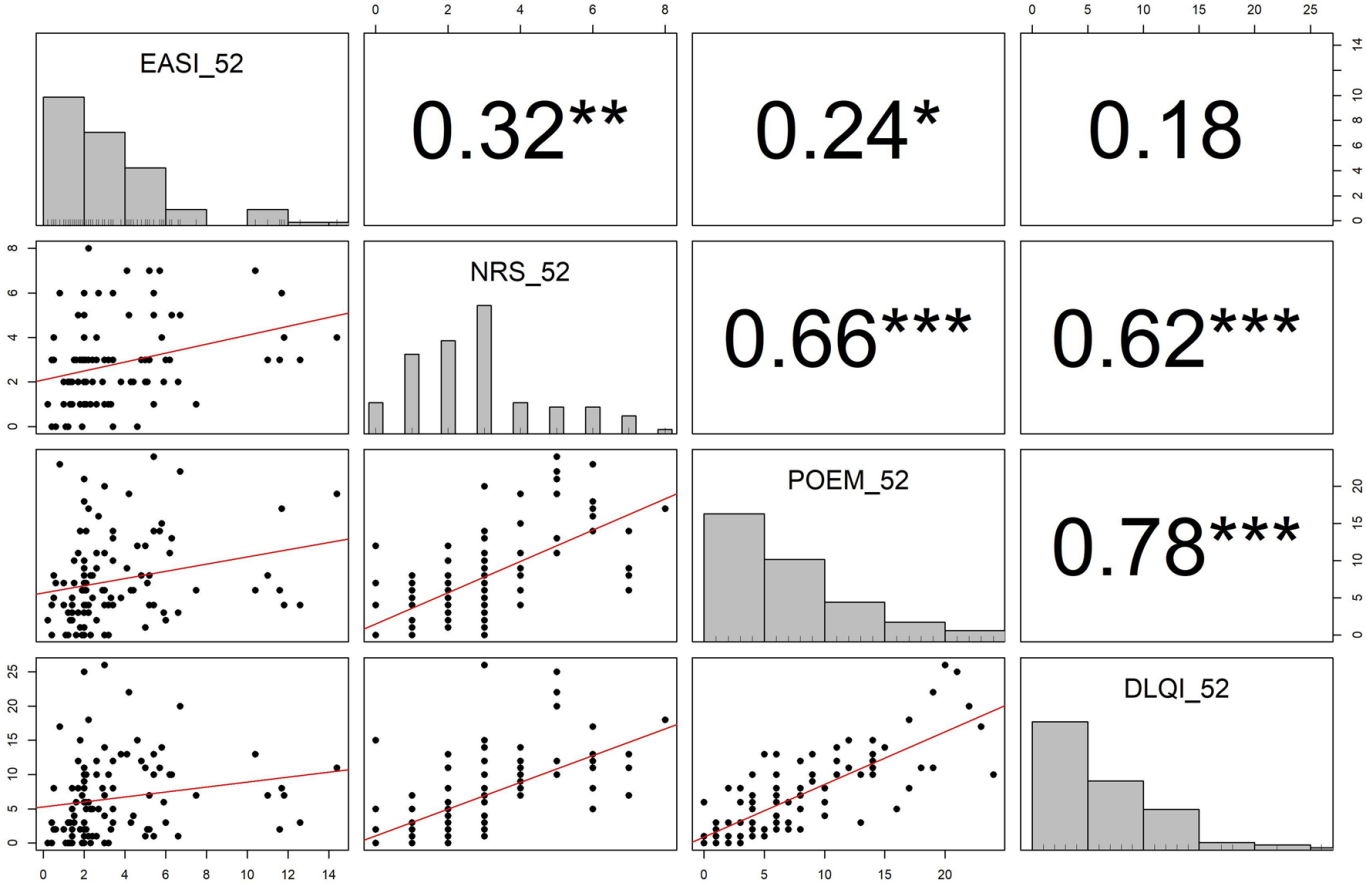
The correlation analysis between EASI, NRS, POEM, and DLQI at 52 weeks. All p-values less than 0.05, 0.01, or 0.001 are summarized as *, **, or ***, respectively.

All variables showed significant correlations, except that between EASI and DLQI. The correlation coefficients of EASI with pruritus NRS, POEM and DLQI were 0.32, 0.24, and 0.18, respectively. On the other hand, about NRS, POEM, and DLQI, which are subjective outcomes compared to EASI, the correlation coefficients between pruritus NRS and POEM, pruritus NRS and DLQI, and POEM and DLQI were 0.66, 0.62, and 0.78, respectively with the correlation coefficient between POEM and DLQI being the highest (Fig. 2).

### Relationship between laboratory markers and treatment response

The improvement in EASI after 52 weeks was significantly lower in the group with high LDH than in the group with low LDH at baseline, 16 and 32 weeks, respectively. Although there were significant differences in each outcome for LDH at 52 weeks, it was difficult to consider the results as statistically appropriate because there was a large difference in sample size between the comparison groups (Table 4). There were no significant differences for IgE and TEC.

**Table 4:** The difference of efficacy, according to LDH—elevated LDH at baseline, 16 and 32 weeks showed inadequate treatment response in EASI.

	LDH at baseline		LDH at 16 weeks		LDH at 32 weeks		LDH at 52 weeks	
	< 250 (n = 30)	≥ 250 (n = 43)	< 250 (n = 66)	≥ 250 (n = 22)	< 250 (n = 63)	≥ 250 (n = 21)	< 250 (n = 65)	≥ 250 (n = 6)
Percent change of EASI after 52 weeks	90.50	85.55	89.42	84.49	90.75	82.49	89.32	78.60
p value*		0.014		0.046		< 0.001		0.052
Percent change of NRS after 52 weeks	70.29	62.63	68.36	57.69	70.46	59.37	69.18	47.22
p value*		0.194		0.067		0.052		0.003
Percent change of POEM after 52 weeks	67.62	66.85	68.76	67.10	71.22	68.06	70.87	36.13
p value*		0.920		0.793		0.576		< 0.001
Percent change of DLQI after 52 weeks	72.78	65.38	70.22	63.33	74.16	61.82	73.49	46.98
p value*		0.315		0.260		0.058		0.013

*p values were obtained by two sample independent t-test.

### Variables associated with achieving EASI 90

Under adjustment to the baseline EASI, we performed logistic regression analysis for each variable. In achieving EASI 90, female sex showed an odds ratio (OR) of 2.509 (p = 0.036) compared to males. About TEC, eosinophilia at the baseline and 16 weeks was not statistically significant, while eosinophilia at 32 and 52 weeks showed OR as 0.250 (p = 0.005) and 0.218 (p = 0.005), respectively. LDH showed statistical significance at all time points, especially at 52 weeks with an OR of 0.068 (p = 0.037) (Table 5).

**Table 5:** The multivariate logistic regression analysis about each variable associated with achieving EASI 90 – female, TEC > 500 at 32 and 52 weeks, and LDH ≥ 250 at all time points showed significant difference, respectively.

Variables	OR	95% CI	P value	Variables	OR	95% CI	P value
Age over 30	0.627	0.275–1.405	0.259	TEC > 500 at baseline	0.621	0.234––1.606	0.330
Sex (female)	2.509	1.077–6.089	0.036	TEC > 500 at 16 weeks	0.477	0.197–1.115	0.093
Onset (adult)	1.004	0.174–5.801	0.996	TEC > 500 at 32 weeks	0.250	0.090–0.641	0.005
Concomitant treatment	0.384	0.114–1.158	0.100	TEC > 500 at 52 weeks	0.218	0.071–0.605	0.005
Concomitant treatment with cyclosporine	0.852	0.378–1.907	0.697	LDH > 250 at baseline	0.259	0.085–0.719	0.012
IgE > 100 at baseline	5.188	0.733–104.917	0.151	LDH > 250 at 16 weeks	0.264	0.079–0.788	0.022
IgE > 100 at 16 weeks	1.186	0.304–4.748	0.804	LDH > 250 at 32 weeks	0.136	0.033–0.446	0.002
IgE > 100 at 32 weeks	0.610	0.175–1.991	0.420	LDH > 250 at 52 weeks	0.068	0.003–0.600	0.037
IgE > 100 at 52 weeks	0.311	0.087–1.023	0.062				

Each analysis was performed about the effect of each variable under adjustment to the baseline EASI, respectively.

In addition, we separately performed multivariative analysis to identify the effects of several variables at once under adjustment to the baseline EASI. It was also confirmed that female sex (OR 3.321, p = 0.033) and high LDH level (OR 0.252, p = 0.036) were factors associated with achieving EASI 90. (Table 6).

**Table 6:** The multivariate logistic regression analysis about variables associated with achieving EASI 90—female and LDH ≥ 250 at baseline showed significant difference.

Variables	OR	95% CI	P value
Age (over 30)	0.708	0.242–2.036	0.522
Sex (female)	3.321	1.141–10.648	0.033
Disease onset (adult onset)	1.456	0.105–18.670	0.765
Concomitant treatment	0.451	0.094–1.871	0.287
Concomitant treatment with cyclosporine	0.802	0.279–2.303	0.680
TEC ≥ 500 at baseline	1.077	0.303–4.062	0.910
LDH ≥ 250 at baseline	0.252	0.064–0.879	0.036

This analysis was performed with the variables presented in the table at once under adjustment to the baseline EASI.

### Adverse events associated with treatment

The main adverse effects associated with dupilumab treatment were facial erythema and conjunctivitis. A total of 19 patients (19.19%) developed facial erythema associated with dupilumab. After dupilumab treatment, 17 patients developed facial erythema, while pre-existing facial erythema became exacerbated in two patients. Most of the facial erythema improved after maintenance with TCI treatment, while one patient who showed no response to TCI treatment improved with oral itraconazole 200 mg once daily for 5 weeks. Six patients developed persistent facial erythema unresponsive to treatment, but considered not serious enough to discontinue dupilumab treatment. One patient was diagnosed with systemic lupus erythematosus (SLE); the erythema improved after increasing the dosing interval of dupilumab and cooperating with the rheumatologist.

Conjunctivitis developed in 17 patients (17.17%) after administration of dupilumab. Most cases improved with increasing the dosing interval or ophthalmic consultation, five patients showed persistent conjunctivitis. None of the patients discontinued from treatment due to conjunctivitis because they thought that the improvement of AD was important and conjunctivitis gradually improved.

## Discussion

While real-world studies on the long-term effectiveness and safety of dupilumab treatment have been conducted and published in various countries^9–14^, there were limited data on its long-term results in Korean AD patients despite our previous study of dupilumab treatment for 16 weeks. In this study, we assessed long-term effectiveness and safety of 52 weeks of continuous treatment with dupilumab in Korean patients with moderate-to-severe AD. In addition, we analyzed the correlation between efficacy outcomes and factors affecting the therapeutic effect^15^. Dupilumab significantly improved clinical factors and symptoms of AD in moderate-to-severe patients over the 52-week treatment period, confirming its long-term therapeutic effect and safety.

Among the baseline values, DLQI was found to be exceptionally high in Korea compared to other studies including that of Japan^8–14^. As mentioned in previous studies, no clear improvements were seen in Korean AD patients although various treatments were used to improve their lesions. This is supported by the relatively high proportion of subjects who used oriental medicine and folk remedy as their past treatments^22^.

Overall, this study showed a higher efficacy than the existing clinical trials and other real-world studies^8–14^; 88.58% of patients achieved significant improvements in EASI at week 52 in this study, compared to 78.3% in LIBERTY AD CHRONOS, and 76.38% in United Kingdom (UK) real-world data. Although outcomes regarding NRS, POEM, and DLQI were similar to those of UK, the results of this study were effective considering that the data from the UK were relatively from mild patients^8,9^. Compared with the results of a study conducted in Japan, our study showed greater efficacy. Particularly, there was a remarkable difference in that the study in Japan resulted in the reduction by 76.5% in EASI after 12 months^14^. In addition, the achievement of EASI 75 and 90 was 90.24% and 53.66% in this study, compared to 65% and 51% in LIBERTY AD CHRONOS or 63% and 29% in the UK, respectively. The following reasons were considered for the higher percentual of improvement in our data to that of existing clinical trials and real-world studies in other countries. Because of the easy accessibility to hospitals in Korea, the majority of patients regularly visited the hospital every two weeks, and the dermatologist could check their condition every time. Whenever their condition worsened, further treatment was immediately initiated. For example, in the case of a patient with a worsening of eosinophilia, which was thought as a poor prognosis, short-term systemic steroid treatment was applied to support the therapeutic effect of dupilumab. In addition to dupilumab treatment, all patients basically used TCI. Patients were fully informed about the importance of using TCI before initiating treatment and continued to use TCI despite the improvements shown with dupilumab. This combination of TCI and dupilumab is thought to increase the therapeutic effect by acting as a “proactive treatment”^23–25^ even after AD has improved. In January 2020, National Health Insurance Service provided coverage for dupilumab treatment in Korea. For insurance coverage, it should satisfy strict conditions with regular dosing intervals and prove the therapeutic effect of dupilumab. If AD worsened, it could no longer be covered by health insurance. Therefore, patients themselves made efforts to use moisturizers or avoid exposure to known allergens; we considered this behavior as “adherence” to the treatment. Based on these results, we could identify the importance of “adherence” and “proactive treatment”.

In the correlation analysis, there was a significantly high correlation between NRS, POEM, and DLQI, which are indicators of subjective symptoms, but there was a low correlation with EASI which is considered as a relatively objective marker. This is consistent with previous studies^15,26^ that suggested the importance of subjective symptoms, in addition to objective symptoms, and particularly shows the usefulness of POEM in evaluating the patient's quality of life.

As suggested in previous studies, our long-term study confirmed that women had a good treatment response, and eosinophilia and high LDH level were associated with a poor treatment response. The real-world study results in which females showed better therapeutic effects than males were also consistent with this study^27^. Compliance and drug distribution according to body weight were considered as the reasons why women have better prognosis. Previous studies have suggested that women have higher treatment compliance than men in AD, contact dermatitis, and psoriasis^28–30^. Therefore, these data may have influenced the ‘adherence’ that appeared during treatment. Although weight and BMI cannot be measured in all patients, if women were assumed to have a lower body weight than men, then the distribution of drugs per unit body weight was higher in females, since the treatment regimen was performed with the same dosage and interval. A clinical trial of dupilumab in children with severe AD also showed a therapeutic effect according to body weight^31^. This is considered meaningful, although it should be verified through validated and controlled trials.

It has been previously suggested that high eosinophilia may be associated with poor therapeutic efficacy of dupilumab^32^. It has also been observed that in these patients, short-term systemic steroids can lower eosinophils and raise the effect of dupilumab. One study suggested that high LDH level is associated with decreased effect of dupilumab under adjustment to the baseline EASI^33^. An ongoing study attempted to investigate the degree of influence of markers known to be related to disease activity in AD on the therapeutic effects of dupilumab treatment. In addition to IgE, TEC and LDH, a total of 18 markers including soluble IL 2 receptor, IL-13, IL-22, IL-31, and thymic stromal lymphopoietin were included^34^. Recently, various therapeutic agents that block each stage of the pathophysiology of AD have been developed^4–6^ and are considered as a good treatment option. Therefore, predictive biomarkers are important as they are used to identify patients who are more responsive to certain therapeutic agents that show good therapeutic effects. In this study, biomarkers that affect the prognosis of dupilumab treatment were identified, which is expected to maximize the effect of dupilumab treatment.

**Figure 3:**
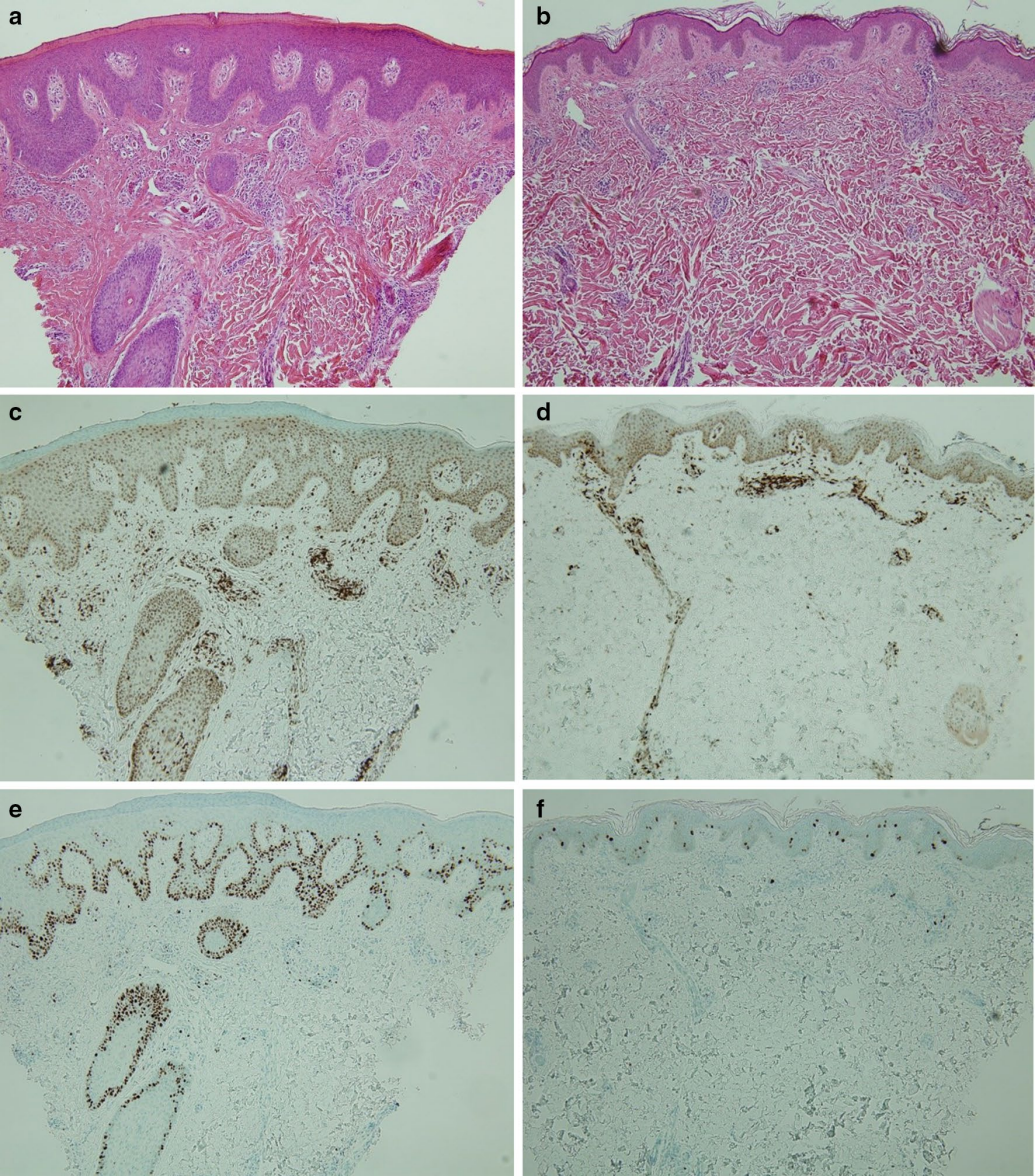
Skin biopsys at baseline and after 16 weeks to be compared by (**a**, **b**) Hematoxylin and eosin, (**c**, **d**) Cluster of differentiation 3 (CD3), and (**e**, **f**) Ki-67. After 16 weeks, the epidermal thickness, irregular acanthosis, the expression of T cells and ki-67 expression were markedly decreased.

Studies about skin biopsy performed before and after dupilumab treatment showed the significant decrease in cytokines associated with type 2 inflammation, such as IL-13 and IL-31, and T cells and dendritic cells, and in epidermal thickness^35,36^. In this study, skin biopsy was performed twice at the same site, before initiating of dupilumab treatment and at 16 weeks (Fig. 3). The epidermal thickness and irregular acanthosis markedly decreased after treatment, and the expression of T cells in the dermis and ki 67, a proliferation marker at the base of the epidermis, was also significantly reduced. Since biopsy was not performed on all patients, increased number of samples in future studies could provide meaningful results for comparison.

Facial erythema is a side effect that has not been specifically reported in existing clinical trials, even though it may have a possible connection with dupilumab treatment in the clinical setting. The cause is not simple; there are various possibilities from exacerbation of existing atopic lesions, rebound by tapering or discontinuation of existing therapeutic agents, allergic contact dermatitis, seborrheic dermatitis positive reaction to Malassezia yeast, to unknown side effects of dupilumab. In rare cases reported, acute cutaneous lupus erythematosus is likely to be stimulated by dupilumab in our SLE patient^37–43^. Therefore, it is necessary to investigate the epidemiologic and clinical characteristics of facial erythema that occurred after dupilumab treatment.

In the previous 16-weeks study^15^, there was a significantly lower rate of conjunctivitis in contrast to the results of existing clinical trials and other real-world studies conducted during the same period. However, the frequency of conjunctivitis increased to a similar degree as in other studies over 16–52 weeks of the treatment period. One study suggested that AD patients treated with dupilumab had an increased risk of developing conjunctivitis^44^. In particular, there was high risk of conjunctivitis in patients with severe AD prior to initiation of treatment, and high frequency in cases of prior conjunctivitis or increased thymus and activation-regulated chemokines, IgE, or eosinophils before initiation of treatment^45–47^. Although the etiology of conjunctivitis in dupilumab-treated patients remains unknown, the following causes are considered. The reduced ocular cytokine due to dupilumab forms an environment which favors the growth of Demodex mites^48^. Eosinophilia after dupilumab treatment^46^, increase in downstream activity of OX40 ligand^49^, inhibition of IL-13 and indirect decreased production of mucin in the goblet cells of the conjunctiva are also potential factors that affect conjunctivitis^50^. In addition, it has been suggested that there is no need to discontinue dupilumab or adjust the interval to control conjunctivitis. Accordingly, patients with prior conjunctivitis are not contraindicated for dupilumab, but ophthalmology treatment is recommended prior to initiation of dupilumab^44^. Conjunctivitis is an important side effect related to dupilumab treatment. Therefore, identification of symptoms by the dermatologist is crucial, and the treatment of conjunctivitis should be properly performed through cooperation with the ophthalmologist.

## Conclusion

Retrospective real-world studies are very important to define the effectiveness of new drugs. In this long term 1 year study including 99 Korean patients, we confirmed the continuous therapeutic benefit of dupilumab, with most patients responding to treatment. We identified potential factors associated with response such as female sex, baseline eosinophilia, and high LDH levels were Additionally, this study confirmed that “adherence” and “proactive treatment” can increase the therapeutic effect of dupilumab in real life. This study is useful in the understanding of long-term dupilumab treatment in Asian patients with moderate to severe AD.
